# Hyperpolarized [1-13C] pyruvate MR spectroscopy detect altered glycolysis in the brain of a cognitively impaired mouse model fed high-fat diet

**DOI:** 10.1186/s13041-018-0415-2

**Published:** 2018-12-18

**Authors:** Young-Suk Choi, Somang Kang, Sang-Yoon Ko, Saeram Lee, Jae Young Kim, Hansol Lee, Jae Eun Song, Dong-Hyun Kim, Eosu Kim, Chul Hoon Kim, Lisa Saksida, Ho-Taek Song, Jong Eun Lee

**Affiliations:** 1 Department of Radiology and Research Institute of Radiological Science, Yonsei University College of Medicine, Seoul, 03722 Republic of Korea; 2 Department of Anatomy, BK21 Project for Medical Science and Research Institute of Radiological Science, Yonsei University College of Medicine, 50-1 Yonsei-ro, Seodaemun-gu, Seoul, 03722 Republic of Korea; 3 Department of Electrical and Electronic Engineering, Yonsei University, Seoul, 03722 South Korea; 4 Department of Psychiatry, Institute of Behavioral Science in Medicine, Yonsei University College of Medicine, Seoul, 03722 Republic of Korea; 5 Department of Pharmacology, Yonsei University College of Medicine, Seoul, 03722 Republic of Korea; 6 BK21 PLUS Project for Medical Sciences and Brain Research Institute, Yonsei University College of Medicine, Seoul, 03722 Republic of Korea; 7 Department of Psychology and MRC/Wellcome Trust Behavioural and Clinical Neuroscience Institute, University of Cambridge, Downing Street, Cambridge, CB2 3EB UK; 8 Molecular Medicine Research Group, Robarts Research Institute & Department of Physiology and Pharmacology, Schulich School of Medicine & Dentistry, Western University, London, ON Canada; 9 The Brain and Mind Institute, Western University, London, ON Canada

**Keywords:** Brain metabolism, Cognitive impairment, High-fat diet, Hyperpolarized ^13^C, Pyruvate metabolism, Magnetic resonance spectroscopy

## Abstract

Higher dietary intakes of saturated fatty acid increase the risk of developing Alzheimer’s disease and dementia, and even in people without diabetes higher glucose levels may be a risk factor for dementia. The mechanisms causing neuronal dysfunction and dementia by consuming high-fat diet degrading the integrity of the blood-brain barrier (BBB) has been suggested but are not yet fully understood, and metabolic state of the brain by this type of insult is still veiled. The objective of this study was to investigate the effect of high-fat diet on the brain metabolism by a multimodal imaging method using the hyperpolarizedcarbon 13 (^13^C)-pyruvate magnetic resonance (MR) spectroscopy and dynamic contrast-enhanced MR imaging in conjunction with the biochemical assay and the behavior test in a mouse model fed high-fat diet (HFD). In mice were fed 60% HFD for 6 months, hyperpolarized [1-^13^C] pyruvate MR spectroscopy showed decreased perfusion (*p* < 0.01) and increased conversion from pyruvate to lactate (*p* < 0.001) in the brain. The hippocampus and striatum showed the highest conversion ratio. The functional integrity of the blood-brain barrier tested by dynamic contrast-enhanced MR imaging showed no difference to the control. Lactate was increased in the cortex (*p* < 0.01) and striatum (*p* < 0.05), while PDH activity was decreased in the cortex (*p* < 0.01) and striatum (*p* < 0.001) and the phosphorylated PDH was increased in the striatum (*p* < 0.05). Mice fed HFD showed less efficiency in learning memory compared with control (*p* < 0.05). To determine whether hyperpolarized ^13^C-pyruvate magnetic resonance (MR) spectroscopy could detect a much earier event in the brain. Mice fed HFD for 3 months did not show a detectable cognitive decline in water maze based learning memory. Hyperpolarized [1-^13^C] pyruvate MR spectroscopy showed increased lactate conversion (*P* < .001), but no difference in cerebral perfusion. These results suggest that the increased hyperpolarized [1-^13^C] lactate signal in the brain of HFD-fed mice represent that altered metabolic alteration toward to glycolysis and hypoperfusion by the long-term metabolic stress by HFD further promote to glycolysis. The hyperpolarized [1-^13^C] pyruvate MR spectroscopy can be used to monitor the brain metabolism and will provide information helpful to understand the disease process.

**Electronic supplementary material:**

The online version of this article (10.1186/s13041-018-0415-2) contains supplementary material, which is available to authorized users.

## Introduction

The metabolic disorder has been suggested as a risk factor to induce cognitive decline and dementia. Moreover, higher dietary intakes of saturated fatty acid increase the risk of developing Alzheimer’s disease and dementia [1, 2]. Patients with diabetes have two-fold risk to develop Alzheimer’s disease and also shorten the conversion time from preclinical to mild cognitive impairment [3, 4]. Interestingly, people with hyperglycemia without diabetes also showed a positive correlation with the cognitive decline and dementia [5, 6]. The mechanisms causing neuronal dysfunction and dementia have been suggested that reduced the tight junction proteins by elevated circulating amyloid-β levels [7] or by inflammation [8], but are not yet fully understood. And the impairment of insulin homeostasis in diabetes has been suggested to accelerate susceptibility to Alzheimer’s disease [9] by activating glycogen synthesis kinase-3, a kinase for tau protein, promote neurofibrillary tangle and beta-amyloid production [10, 11]. But the metabolic state of the brain affected by this type of insult is still veiled and imaging method to quantitatively present the metabolic information in the brain at the earlier process related to cognitive decline and dementia is needed.

A Fluorine 18 (^18^F) fluorodeoxyglucose (FDG) positron emission tomography (PET) study in Alzheimer’s disease reported decreased cerebral glucose metabolism along with amyloid-β accumulation using the ^11^C-Pittsburgh compound B PET imaging [12]. A decreased state of glucose metabolism was thought to be an early marker of dementia before diagnosed with cortical atrophy or clinical symptoms [13]. On the other hand, spatial correlations between the sites of active aerobic activity in young adults and those of beta-amyloid deposits in the elderly have been reported by Pittsburgh compound B and FDG-PET imaging studies [14]. Therefore, it is unclear whether any abnormal glucose metabolism affects the early stages of cognitive impairment. An FDG-PET study of Alzheimer’s disease showed 89% diagnostic accuracy in the reduction of the cerebral metabolic rate in the brain [15]. However, serum glucose levels above 160 mg/dL limit the use of brain ^18^F-FDG, and a systematic review has shown that standardized uptake value in the brain is inversely proportional to glycemia [16, 17]. Therefore, it is necessary to have an imaging method that can observe the metabolism in the brain without being affected by blood sugar.

Hyperpolarized carbon 13 (^13^C) magnetic resonance (MR) spectroscopy can detect in vivo metabolism by 10,000-fold increased sensitivity using ^13^C enriched endogenous metabolic substrates without being exposed to ionizing irradiation [18, 19]. Hyperpolarized [1-^13^C] pyruvate MR spectroscopy can detect [1-^13^C] lactate catalyzed by lactate dehydrogenase (LDH), ^13^C-alanine by alanine aminotransferase, and ^13^C bicarbonate by pyruvate dehydrogenase (PDH) [20]. The purpose of the present study was to assess the brain metabolism by using multimodal imaging method including hyperpolarized [1-^13^C] pyruvate MR spectroscopy in conjunction with the biochemical assay and the behavior test in a cognitivelyimpaired a mouse model fed a high-fat diet (HFD).

## Material and methods

### Animal procedures

**Fig. 1:**
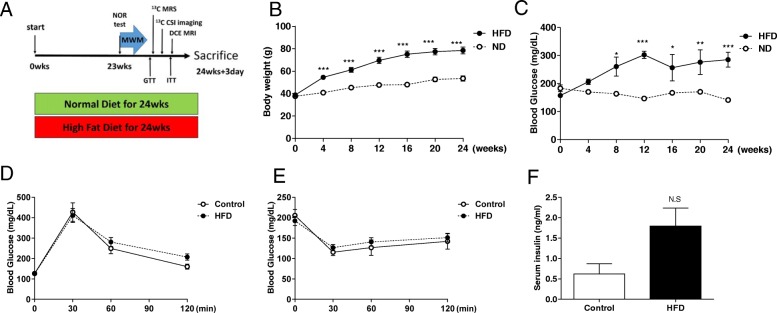
High-fat diet (HFD) fed mice gained weight and presented hyperglycemia. (**a**) Experimental schedule. (**b**) Body weight was measured every 4 weeks on each diet group. (**c**) Fasting serum glucose level. (**d**) Glucose tolerance test. (**e**) Insulin tolerance test. (**f**) Serum insulin level measured using ELISA in the 24th week of the diet (*p* = 0.074). The *p*-values were obtained from the two-tailed Student’s t-test between HFD-fed mice and control groups (*n* = 10 for both groups) *** *p* < 0.001. Abbreviation: BW = body weight; GL = serum glucose level; NOR = novel object recognition test; MWM = water maze behavior test; ^13^C MRS = ^13^C magnetic resonance spectroscopy; ^13^C CSI = ^13^C chemical shift image; DCE-MRI = dynamic contrast-enhanced magnetic resonance imaging

Male ICR mice (30–35 g, seven weeks-old) were purchased from Japan SLC, a branch of Charles River Laboratories (Shizuoka, Japan). Mice were fed either a normal diet (ND, 5053, PicoLab, 13.1 kcal % fat; control mice) or High Fat diet (HFD, D12492, Research Diet INC., Fat 54.3% kcal of lard, 5.6 kcal of soybean oil) for 12 weeks and 24 weeks (Table 1). The experimental schedule of 12 weeks and 24 weeks were represented in Fig. 1a and 7a, respectively.

**Table 1:** Diet composition

	Normal diet	High-fat diet
Protein (kcal %)	24.5	20
Carbohydrate (kcal %)	62.4	20
Fat (kcal %)	13.1	60

Rodent Diet with 60 kcal% Fat (D12492, Research Diets INC,.) Protein: 20% kcal; Protein (Casein, Lactic, 30 Mesh), Protein (Cystine, L), Fat: 60% kcal; Fat (Lard), Fat (Soybean Oil, USP), Carbohydrate: 20% kcal; Carbohydrate (Lodex 10), Carbohydrate (Sucrose, Fine Granulated), Fiber (Solka Floc, FCC200), Mineral (S10026B), Vitamin (Choline Bitartrate, V10001C), Dye (Blue FD&C #1, Alum. Lake 35 ~ 42%), Energy Density: 5.21 kcal/g

All animal procedures were carried out according to the protocol approved by the International Animal Care and Use Committee (IACUC) of the Yonsei University Animal Research Center (YLARC, permission No. 2015–0039) following NIH guidelines. All animals were maintained in a specific pathogen-free facility of the YLARC with well controlled temperature (23 °C) and Light cycle (12 h light and 12 h dark) and easy access to water and food.

### Determination of body weight and serum glucose levels

Body weight (BW) and fasting serum glucose levels of all animals were monitored. To measure fasting glucose levels, mice fasted for 4 h before the test. Blood glucose concentrations from blood samples taken from the tip of the tail were measured using a glucometer. The body weight and glucose levels were performed every 4 weeks.

### Intraperitoneal glucose tolerance test (IPGTT)

Glucose tolerance test is a widely used to diagnose glucose intolerance in obesity and type II diabetes mellitus [21, 22]. The intraperitoneal glucose tolerance test was performed at 24 weeks after high fat diet. Food was removed a night before the test. The mice were injected with glucose (1 g/kg/ip, dissolved in saline) in the morning. Blood glucose levels from blood samples taken from the tail vein were measured using a glucometer at 0, 30, 60, and 120 min after the bolus [11].

### Intraperitoneal insulin tolerance test (IPITT)

The intraperitoneal insulin tolerance test was performed three days later after finishing IPGTT at 24 weeks after high fat diet. Mice fasted for 4 h before the test. The mice were injected with human recombinant insulin (0.75 unit/kg/ip, dissolved in saline). Blood glucose levels from blood samples taken from the tail vein were measured using a glucometer at 0, 30, 60, and 120 min after the bolus [11].

### Serum insulin ELISA

The serum insulin ELISA test was performed at 24 weeks after high fat diet. Mice were sacrificed and the blood was collected through cardiac puncture for EDTA-plasma preparation. Serum insulin was measured using an insulin ELISA kit (ALPCO, Windham, NH, USA). 10 μL of each standard and control samples were loaded into appropriate wells. Then, 75 μL of enzyme conjugate (mouse monoclonal anti-insulin conjugated to biotin) was added to each well and incubated for 2 h at room temperature on the microplate shaker at 800 rpm. After washing the microplate six times with 350 μL of wash buffer, 100 μL of substrate solution, tetramethylbenzidine, was added to each well and incubated for 15 min at room temperature on the microplate shaker. The enzymatic reaction was stopped by adding 100 μL of stop solution to each well, and the absorbance was measured at 450 nm using a microplate reader.

### Hyperpolarized ^13^C MR spectroscopy

We used 26.7 mg of [1-^13^C] pyruvic acid (Cambridge Isotope, Tewksbury, MA) mixed with 15 mM trityl radical OX-063 (Oxford Instruments, Oxford, UK) and 0.75 mM gadoteratemeglumine (Dotarem®, Guerbet, France) for hyperpolarized ^13^C MRS. We hyperpolarized the sample using a dynamic nuclear polarization system (HyperSense®, Oxford Instruments, Oxford, UK) and dissolved it with 3.8 mL of Tris/EDTA-NaOH buffer, resulting in 79 mM pyruvate (pH 7.5) with a polarized level of approximately 20%. We drew 350 μL of hyperpolarized [1-^13^C] pyruvate into a syringe for in vivo MR spectroscopy.

We performed in vivo hyperpolarized MR spectroscopy using a 9.4 T animal imaging system (BioSpec 94/20, Bruker BioSpin MRI GmbH, Ettlingen, Germany) with a ^1^H-^13^C dual-tuned surface transmit/receive coil. We acquired time-resolved ^13^C free induction decay data from 7.5 mm axial slices of the whole brain with a flip angle of 10° and time resolution of 1 s by using a pulse-and-acquire sequence [23]. For the mapping of metabolites, a single time point hyperpolarized ^13^C free induction decay chemical shift image was obtained using centric-ordered phase encoding with a flip angle of 10° from 3.5 mm coronal slices of the brain using a ^13^C single tune mouse head coil. Field of view was 18 × 24 mm^2^ with a matrix size of 18 × 24 or 9 × 12. We produced a hyperpolarized ^13^C metabolite map by measuring the peak value of each metabolite and overlaid it on the proton T2 weighted image. The images were acquired for 35 s from 18 s after intravenous injection of pyruvate. All data were processed using MATLAB-based analysis (R2017a, MathWorks, Natick, MA, USA).

### Dynamic contrast-enhanced MR imaging

We performed dynamic contrast-enhanced MR imaging on a 3 T system (Discovery™ MR750, GE Healthcare, WI, USA) to evaluate the integrity of the blood-brain barrier function [24]. Pre- and post-contrast T1-weighted images were acquired by injecting 0.2 mmol/kg gadoteratemeglumine (Doctarem®, Guerbet, Villepinte, France) into the tail vein. Data were transferred to a workstation and analyzed using GenIQ software (GE Medical Systems, WI, USA).

### Cognitive function test

We performed a Morris water maze test and object-location memory test to evaluate the cognitive function as previously described [25]. Briefly, the Morris water maze test measured the time required to reach the hidden platform and escape-latency in a circular pool 90 cm in diameter and 30 cm in depth. The pool has quadrants by four different visual cues, and a hidden platform 12 cm in diameter submerged 2 cm below the black water surface in one of the quadrants. In location memory task, the experiment was performed in a black, rectangular, acrylic open field box (25 cm sides) with 3-dimensional plastic visual cue placed on the edge of the area. Mice were allowed to explore the open field box with no objects but internal cue on one of the walls for 10 min for two consecutive days. Twenty-four hours later, the trial was performed. Two identical plastic objects were placed in two opposite corner of the internal cue wall, where the mice were allowed to freely explore the objects for 10 min. Another twenty-four hours later, the test was performed in the same box, where one of the objects was moved to the novel location of the arena. The movements of the mice were video-recorded for 5 min. All objects and arena were cleaned using 30% Ethanol between every trial. Time spent for touching the objects using nose was measured (T novel: time spent for touching the object placed in the novel location; T familiar: time spent on touching the object to the familiar location). Preference for the object displaced to the novel location was calculated as the percent time. Discrimination index was calculated with the formulation of [(T_novel_– T_familiar_)/(T_novel_ + T_familiar_)].Video recording was performed using an Ethovision system (Noldus, Wageningen, The Netherlands).

### Assessment of PDH activity

PDH activity was measured using an assay kit (Abcam, Cambridge, UK). Samples (200 μL) were incubated for 3 h at room temperature. The microplate was washed twice with 300 μL of stabilizer, and then 200 μL of assay solution was added. The absorbance of each well was measured at 450 nm using a kinetic program for 15 min with a microplate reader.

### Assessment of lactate level

Lactate levels were measured using the L-Lactate assay kit (Abcam, Cambridge, UK). Extracted blood from the euthanized mice was centrifuged at 15,000 g for 5 min at 4 °C to separate serum. Tissue samples were harvested and lysed using an NP-40 buffer. After measuring BCA-based protein concentration, 40 μg of lysate was used to detect lactate concentration. The absorbance was measured at 450 nm according to the manufacturer’s protocol.

### Western blot analysis

The collected hippocampal, neocortical and striatal tissues were homogenized in ice-chilled 20 mM pH 7.4Tris-HCl buffer. Homogenate containing 15 μg of protein was subjected to 8% SDS-PAGE under reducing conditions. The proteins were transferred to PVDF membranes in transfer buffer and then separated at 400 mA for 2 h at 4 °C. The Western blots were subsequently incubated for 2 h with 5% skim milk at room temperature and then incubated overnight with a 1:1000 dilution of anti-LDHA (NBP1–48336; NovusBio, CO, USA), anti-β-actin (sc-47,778; Santa Cruz Biotechnology, TX, USA), anti-LDHB (AB85319; Abcam, Cambridge, UK), anti-claudin5 (ab-15,106; Abcam, Cambridge, UK), anti-p-PDH (ab-92,696; Abcam, Cambridge, UK) and anti-PDH antibodies (9H9AF5; The Thermo Fisher Scientific, MA, USA). Then, the blots were washed twice with Tween 20/Tris-buffered saline (TTBS) and incubated with a 1:3000 dilution of horseradish peroxidase-conjugated secondary antibody for 2 h at room temperature. After washing 3 times with TTBS, blots were developed using enhanced chemiluminescence (Amersham Life Science, Arlington Heights, IL, USA). The membranes were analyzed using the Multi Gauge bioimaging program on the Las-4000 mini (Fujifilm Life Science USA, Stamford, CT, USA).

### Statistical analysis

Data were analyzed using a one-way analysis of variance (ANOVA) followed by Newman-Keuls test for post-hoc comparisons. Student’s t-test was used to compare the two groups. In the behavioral study, data were analyzed using a two-way ANOVA followed by Bonferroni’s test for post-hoc comparisons. Dynamic conversion ratio was analyzed using a linear mixed model with random analysis. All results were expressed as a mean ± standard error of the mean, and *p* < 0.05 was considered statistically significant. Statistical analysis was performed by using statistical software (PRISM version 6.0, GraphPad Software, CA, USA; SPSS 23, SPSS Inc., IL, USA).

## Results

### Mice fed HFD for 6 months showed higher lactate conversion in hyperpolarized ^13^C MRS

**Fig. 2:**
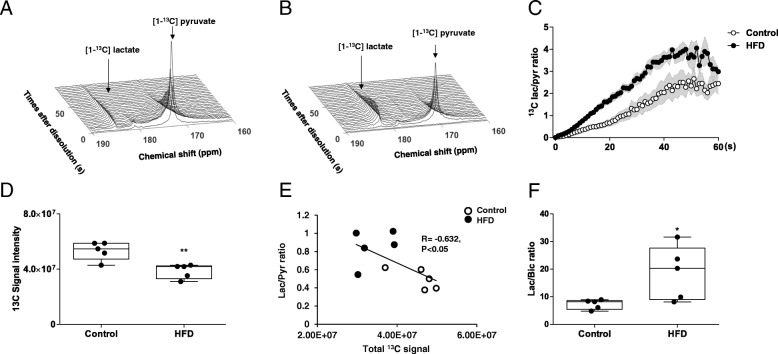
HFD-fed mice showed increased lactate signal and decreased brain perfusion in hyperpolarized ^13^C magnetic resonance (MR) spectroscopy. *A*, *B*, The stack plot of sequential spectra collected every second displayed for 90 s of the hyperpolarized ^13^C MR spectrum shows [1-^13^C] pyruvate at 173 ppm and [1-^13^C] lactate at 185 ppm in the brain of control (**a**) and HFD-fed mice (**b**). (**c**) The dynamic conversion ratio of hyperpolarized [1-^13^C] pyruvate/[1-^13^C]lactate calculated from the dynamic peak intensities (*p* < .0001). Shaded regions represent standard error of the mean value (*n* = 5 for both groups). (**d**) The box plot shows the total hyperpolarized ^13^C signal from the brain obtained for 10 s after the injection (*P* < .01). (**e**) Hyperpolarized [1-^13^C]lactate/[1-^13^C]pyruvate ratio showed a negative correlation with total ^13^C signal (*n* = 10, Pearson’s *r* = − 0.632, *P* < .05). (**f**) The ratio of [1-^13^C] lactate/^13^C-bicarbonate calculated from the peak intensity (*n* = 5 for both groups). Error bars represent standard error of the mean. * *p* < 0.05

Mice fed HFD for 24 weeks showed hyperglycemic state with weight gain represented by an increased fasting glucose level when compared with normal diet fed mice. However, no difference was observed in the glucose tolerance test, insulin tolerance test, and serum insulin level (*n* = 10 for both groups; *p* < 0.001; Fig. 1b-f). We have investigated the metabolic influence of the hyperglycemic state in the brain of 24 weeks after HFD fed mice using by the hyperpolarized ^13^C MR spectroscopy. Which were detected [1-^13^C] pyruvate at 173 ppm, and [1-^13^C] lactate at 185 ppm in the brain of control (Fig. 2a) and HFD-fed mice (Fig. 2b). The dynamic conversion ratio of hyperpolarized [1-^13^C] lactate/[1-^13^C] pyruvate calculated from the peak intensities of the MR spectrum showed significantly increased lactate conversion in the brain of HFD-fed mice (*n* = 5 for both groups, *p* < 0.0001; Fig. 2c). HFD-fed mice showed significantly decreased total ^13^C signal in the brain, which represents perfusion, calculated by the sum of the area under the spectrum for 10 s from the injection (41.4 ± 7.6 vs. 28.8 ± 4.28 × 10^7^, respectively; n = 5 for both groups; *p* < 0.01; Fig. 2d). Hyperpolarized [1-^13^C] lactate/[1-^13^C] pyruvate ratio showed a negative correlation with total ^13^C signal (Fig. 2e; *n* = 10, Pearson’s *r* = − 0.632, *p* < 0.05). [1-^13^C] pyruvate could estimate the mitochondrial metabolism, because pyruvate converted to acetyl Co-A and CO_2_ by PDH in the mitochondria. Therefore, hyperpolarized ^13^C bicarbonate, in equilibrium with CO_2,_ directly reflects the TCA cycle rate [26]. To evaluate metabolic preference between cytoplasmic glycolysis and mitochondrial oxidation, we analyzed [1-^13^C] lactate/^13^C-bicarbonate ratio. Hyperpolarized [1-^13^C] lactate/^13^C-bicarbonate ratio was increased in HFD-fed mice (n = 5, for both groups, *P* < 0.05; Fig. 2f).

Rate constants converting pyruvate to lactate (KP) by lactate dehydrogenase (LDH) catalyzed reaction was calculated by fitting the peak intensities of pyruvate and lactate to the modified Bloch equations for two-site exchange as previously described [27]. KP for control and HFD-fed mice were 0.021 ± 0.009 and 0.056 ± 0.015, respectively. These results represented that brain metabolism in the mice fed HFD activated cytosolic glycolysis in the mice fed HFD for 6 months.

**Fig. 3:**
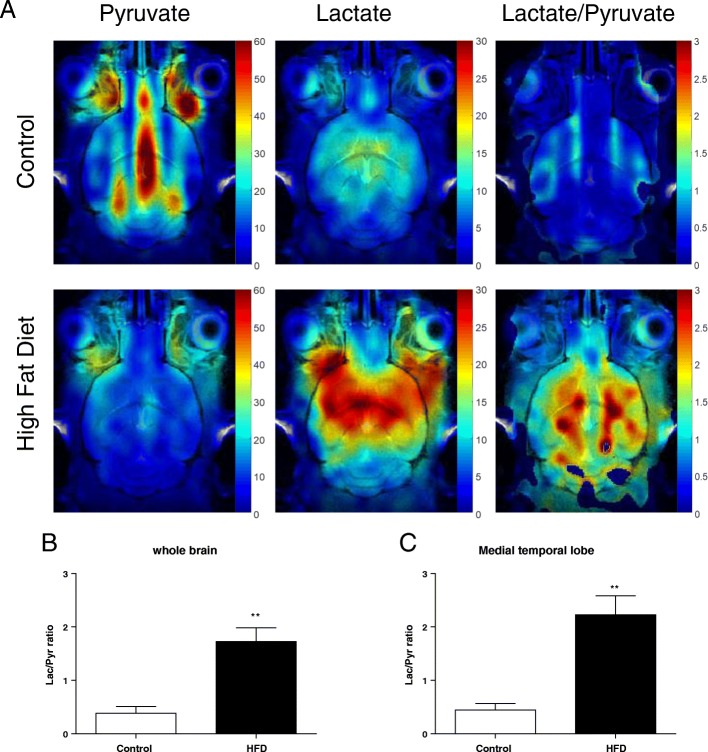
Chemical shift imaging of hyperpolarized ^13^C MR spectroscopy. (**a**) Color maps overlaid on the ^1^H images represent [1-^13^C] pyruvate and [1-^13^C] lactate peak intensities, and[1-^13^C] lactate/[1-^13^C] pyruvate intensity ratios. The images were acquired for35s from 18 s after intravenous injection of 79 mM hyperpolarized ^13^C-pyruvate in the coronal plane with 3.5 mm slice thickness and 1 × 1 mm^2^ in-plain resolution. (**b**) [1-^13^C] lactate/[1-^13^C] pyruvate intensity ratios in the whole brain. (**c**) [1-^13^C] lactate/[1-^13^C] pyruvate intensity ratios in the Medial temporal lobe

**Fig. 4:**
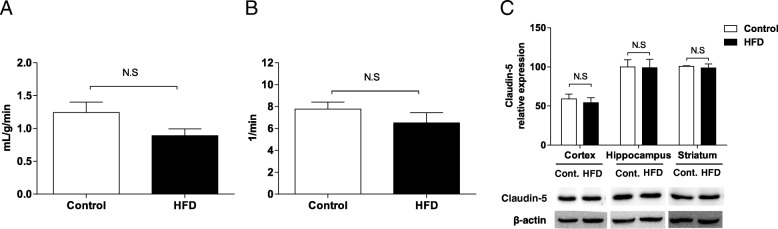
Intact blood-brain barrier function in HFD fed mice. (**a**) Transfer constant, (**b**) rate constant, and (**c**) cropped images of claudin-5 and the quantified claudin-5 were by the ratio to the β-actin showed no difference (*n* = 3–4 for both groups)

The metabolite map of the brain was explored using hyperpolarized ^13^C chemical shift imaging (*n* = 4–5 for both groups, Fig. 3a). The [1-^13^C] pyruvate perfusion signal was mainly seen in veins in the retro-orbital area, sagittal sinus, and transverse sinus of control mice, but the parenchymal [1-^13^C] lactate signal was weak. On the other hand, although the [1-^13^C] pyruvate perfusion signal was weak, the [1-^13^C] lactate metabolite signal was strongly seen in the brain parenchyma of HFD-fed mice. The highest [1-^13^C] lactate/[1-^13^C] pyruvate conversion ratio was detected in the hippocampus and striatum. Voxel based analysis represented that higher [1-^13^C] lactate/[1-^13^C] pyruvate conversion was not only in the brain (Fig. 3b), but also in medial temporal lobe (Fig. 3c). The blood-brain barrier (BBB) permeability could influence [1-^13^C] pyruvate delivery to the brain. Thus we assessed permeability in the brain of mice fed HFD using dynamic contrast-enhanced (DCE)-MRI and calculated the transfer constant (K_trans_) from blood plasma into the extravascular-extracellular space and rate constant (K_ep_) from extravascular-extracellular space back to the blood plasma. DCE MRI showed no differences in the calculated permeability parameters, transfer constant (Fig. 4a) and rate constant (Fig. 4b) (*n* = 3, for both groups). Also the expression level of claudin-5, a blood-brain barrier integral protein, was not different (n = 3, for both groups; Fig. 4c).

### Mice fed HFD for 6 months showed decreased PDH activity and increased lactate production

**Fig. 5:**
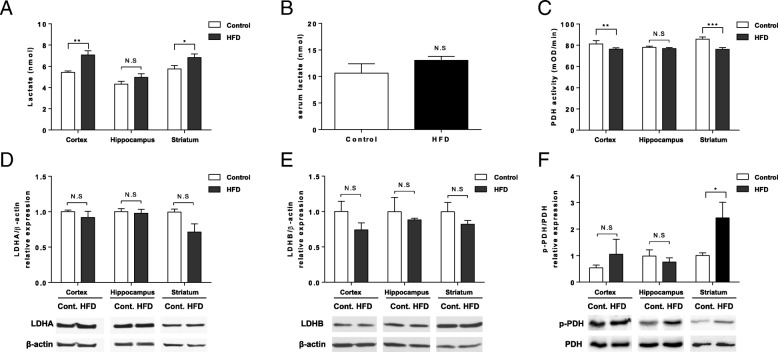
Increased lactate production and decreased pyruvate dehydrogenase (PDH) activity in HFD fed mice. (**a**) Amount of lactate in 40 μg of a lysate of cortex, hippocampus and striatum tissues (*n* = 5 for both group). (**b**) Serum lactate level measured using ELISA (10.64 ± 1.745 vs. 13.02 ± 0.75; *n* = 10 each). (**c**) PDH activity measured in the cortex, hippocampus and striatum tissues (*n* = 5–6 for both groups). (**d**) Quantified LDHA by the ratio to β-actin and cropped images (*n* = 5 for both groups). (**e**) Quantified LDHB by the ratio to β-actin and cropped images (*n* = 5 for both groups). (**f**) Quantified Phosphorylated PDH by the ratio to total PDH and cropped images (*n* = 5 for both groups). * *p* < 0.05, ** *p* < 0.01, *** *p* < 0.001

Since the signal intensity of hyperpolarized [1-^13^C] lactate reflect the amount of lactate pool in the tissue [27], we measured the lactate content in the brain cortex, hippocampus, and striatum. The amount of lactate significantly increased in the brain cortex (*p* < 0.01), and striatum (*p* < 0.05) in HFD-fed mice (*n* = 5 for both groups, Fig. 5a). However, the serum lactate level showed no difference (Fig. 5b). To elucidate the cause of higher lactate production in the brain tissue we investigated the LDH which catalyzes the reaction between pyruvate and lactate, and pyruvate dehydrogenase (PDH), the first step enzyme for pyruvate oxidation in mitochondria. PDH enzyme activity was decreased in the cortex (*p* < 0.01) and striatum (*p* < .001) (n = 3 for both groups, Fig. 5c). But, the expression level of A and B subunits of LDH in the brain tissue showed no difference (n = 5 for both groups, Fig. 5d,e), and phosphorylated PDH (Ser293) level was increased in the striatum of mice fed HFD (n = 3 for both groups, *p* < 0.05; Fig. 5f).

### Mice fed HFD for 6 months developed cognitive impairment

**Fig. 6:**
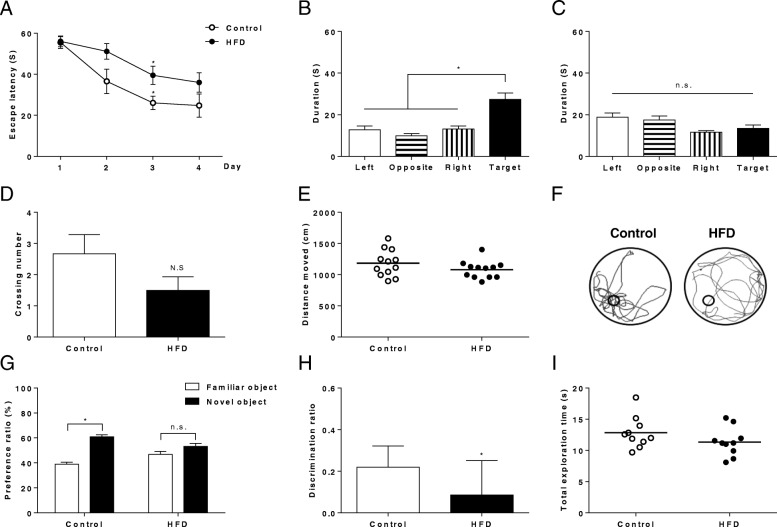
Mice fed HFD showed cognitive impairment. (**a**) Escape latency is the spending time for the mice to find the submerged platform during training days. HFD-fed mice showed impaired spatial learning memory function compared to controls. The time spent in the respective quadrant searching the platform at the probe test for the control group (**b**) and HFD-fed group (**b**). Control mice spent significantly more time in the target quadrant. (**d**) Representative swim paths during probe trial. (**e**) The crossing number of the platform location. (**f**) Total distance moved during the probe test. (**g**) Preference for the object which is displaced to a novel location as the percent time. (**h**) Discrimination index = [(Tnovel– Tfamiliar)/(Tnovel+ Tfamiliar)]; Tnovel, time spent on exploring the novel object; Tfamiliar, time spent on exploring the familiar object. (**i**) Total exploration time. Error bars represent standard error of the mean. *p*-values were obtained from two-way ANOVA with Bonferroni’s post-hoc test (**a, g**), from one-way ANOVA followed by Newman-Keuls post-hoc test (**b**, **c**), and from the two-tailed Student’s t-test to compare two independent groups (**d, e, h i**). (*n* = 10 for both groups, * *p* < 0.05)

Since the hippocampus is the most vulnerable area in subjects with dementia, we performed two hippocampus-dependent cognitive behavior test. In the Morris water maze task, mice were allowed to learn the location of the invisible platform for 4 consecutive days. Although mice fed both control and HFD groups were successful to learn the location of the hidden platform during 4-day trials, the mice fed HFD showed less efficiency in learning the spatial memory (Fig. 6a). Furthermore, the mice fed HFD spent equivalent time in all quadrants with no significant differences during probe test, while mice fed ND explored the target quadrant more than other areas, which implies that mild cognitive impairment can be developed by high fat diet regimen in a mild way (Fig. 6b, c). Therefore, to analyze the behavior patterns of mice fed HFD sensitively, we calculated the platform crossing number during the probe test. The HFD-fed mice showed a decrement in crossing number (Fig. 6d). No difference between total distances moved indicated that HFD did not effect on locomotor activity or motivation (Fig. 6e, f). The object location recognition task assesses cognition, specifically spatial memory and discrimination in rodent models of CNS disorders. Mice fed HFD showed significantly impaired performance in the object location recognition task. The lack of differences in preference ratio and significantly low discrimination ratio were observed in mice fed HFD (*n* = 10 for both groups, *p* < 0.05; Fig. 6g, h). The result of object recognition test might have been confounded by several factors such as anxiety, nomophobia, and motivation or interest of mice in interacting with objects used. However, this is unlikely for we conducted 3-days of habituation, which might minimize mice’s anxiety, and also we found no group difference in exploration time (Fig. 6i), which indicates general motivation to explore objects. To estimate the relation between brain metabolism with the congitive decline, we analyzed the correlation between hyperpolarized [1-^13^C] lactate/pyruate ratio in the medial temporal lobe and time to spent in target qurdrant during 60 s in water mazed behavior test. Hyperpolarized [1-^13^C] lactate/[1-^13^C] pyruvate ratio showed a negative correlation with time to spent in the target quadrant (Additional file 1: Figure S1; *n* = 5, Pearson’s *r* = − 0.692, *p* < 0.05), which implies that incrased glycolysis was associated with cognitive decline.

### Mice fed HFD for 3 months showed increased lactate conversion in hyperpolarized ^13^C MRS without cognitive decline

**Fig. 7:**
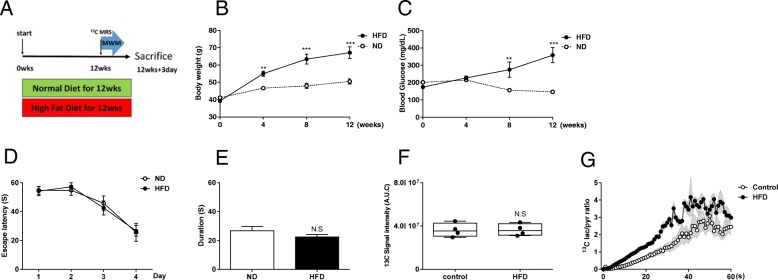
Mice fed HFD for 3 months showed increased lactate conversion in hyperpolarized 13C MRS without cognitive decline. (**a**) Experimental schedule. (**b**) Body weight was measured every 4 weeks on each diet group. (**c**) Fasting serum glucose level. (**d**) Escape latency is the spending time for the mice to find the submerged platform during training days. Escape latency had no significant difference between ND and HFD group. (**e**) The time spent in the target quadrant searching the platform at the probe test for each group. ND and HFD group show no significance in exploration time in the target quadrant. (*n* = 10 for both groups, * *p* < 0.05) (**f**) The box plot shows the total hyperpolarized ^13^C signal from the brain obtained for 10 s after the injection and there was no difference. (**g**) The dynamic conversion ratio of hyperpolarized [1-^13^C] pyruvate/[1-^13^C]lactate calculated from the dynamic peak intensities (*p* < .0001). Shaded regions represent standard error of the mean value (*n* = 5 for both groups)

To determine metaboic alteration toward glycolysis by HFD occur before the cognitive decline, we performed hyperpolarized ^13^C MR spectroscopy in the brain of mice fed HFD for 3 months. They showed significant weight gain (*p* < 0.001, Fig. 7b) and higher fasting serum glucose level to the control mice (n = 5 for both groups; *p* < 0.001; Fig. 7c). In the Morris water maze task, both control and HFD groups did not show the difference to learn the location of the hidden platform during 4-day trials (n = 5 for both groups; Fig. 7d,e). In hyperpolarized [1-^13^C] pyruvate MR spectroscopy, ^13^C signal in the brain, as an indicator of cerebral perfusion, did not distinguish between control and mice fed HFD (Fig. 7f), but the dynamic conversion ratio of hyperpolarized [1-^13^C] lactate/[1-^13^C] pyruvate showed significantly increased in the brain of HFD-fed mice (*n* = 4 for both groups, *p* < 0.001; Fig. 7g), suggesting that increased glycolysis occur before cerebral hypoperfusion and cognitive decline by HFD.

## Discussion

In this work, we presented the early change of the pyruvate metabolism in the brain of an animal model fed HFD. Increased glycolysis may cause an increased hyperpolarized [1-^13^C] lactate signal. Since the increased hyperpolarized [1-^13^C] lactate/[1-^13^C] pyruvate signal ratio could represent not only the state of low oxygen tension [28, 29], but also the increased cytosolic glycolysis without oxygen tension so called anaerobic glycolysis. The perfusion and the metabolic conversion are the significant factors affecting the degree of the hyperpolarized^13^C-lactate signal [28]. As the total ^13^C signal can be an indicator of perfusion [30], decreased total carbon signal corresponds to decreased cerebral perfusion. Reduced perfusion state of the brain fed HFD for 6 months in this study is consistent with a report of decreased perfusion state in Alzheimer’s disease patients [31]. Interestingly, these mice fed HFD for 3 months showed increased hyperpolarized [1-^13^C] lactate conversion without hypoperfusion. On the other hand, mice fed HFD for 6 months showed decreased cerebral perfusion and a negative correlation between the perfusion and the hyperpolarized [1-^13^C] lactate/[1-^13^C] pyruvate ratio. Those results suggest that increased glycolysis may be an earlier metabolic alteration and cerebral hypoperfusion by long-term exposure to HFD may further promote to be converted to lactate as a consequence of tissue hypoxia [32].

Recently an MR spectroscopy study using [1-^13^C] glucose reported an age-dependent change of glucose metabolism in a triple transgenic (3xTG) Alzheimer’s disease mouse model. In 7-month mice, brain metabolism increased, while it decreased in 13-month mice [33, 34]. According to the FDG-PET study in this 3xTG mice, FDG uptake significantly decreased in the almost the whole brain of 18-month mice, but decreased in the special region containing cingulate gyrus of 12-month mice [35]. Those results suggest that alteration toward to glycolysis may be an earlier metabolic event than decreased glucose metabolism shown in FDG-PET imaging and therefore hyperpolarized [1-^13^C] pyruvate MR spectroscopy have a potential to monitor earlier disease process.

Hyperpolarized ^13^C MR spectroscopy showed the highest [1-^13^C] lactate/[1-^13^C] pyruvate signal ratio not only in the hippocampus known as the particularly affected in Alzheimer’s disease [36], but also in stratum in mice fed HFD for 6 months. Memories of hippocampal and striatal systems are thought to operate independently and to support place-based learning under the control of the hippocampus, and response-based learning under the control of the striatum [37]. On the other hand, a report showed impairment of place learning memory in the dorsomedial striatal injury [38]. The other report using water maze based spatial memory test showed dorsomedial striatum was activated during early learning and getting inactivated during late learning, and this pattern was also observed in human [39], suggesting the importance of striatum in learning memory.

Studies on the relationship between lactate level and cognition in the brain have been reported. Lactate amount in frontal cortex and interstitial fluid of the hippocampus was elevated in APP/PS1 transgenic mice having cognitive decline [40]. In human studies, increased lactate level was reported in the cerebrospinal fluid of Alzheimer’s disease patients [41], and it showed a negative correlation with memory performance in individuals with mild cognitive impairment [42]. Furthermore, it has been reported that acute hyperglycemia increased lactate and amyloid beta in the hippocampal interstitial fluid and that suggest increased glucose metabolism regulates neuronal activity via KATP channel in APP/PS1 mice [43]. However, it is mostly unknown whether enhanced lactate production is beneficial or harmful to memory function.

In early onset Alzheimer’s disease, genetic factors such as amyloid precursor protein, or preseniline(PSEN) 1 or 2 has been regarded as dominant factors, but in late onset Alzheimer’s disease(LOAD) environmental factor such as metabolic disease has been regarded to induce Alzheimer’s pathogenesis. Genetically in LOAD, the apolipoprotein E(APOE) gene is the strong factor to cognitive decline. APOE gene has the three polymorphism- ε2, ε3, and ε4. Among them, almost 40% of patients having Alzheimer’s disease have ApoE ε4 alleles [44]. According to the animal study, mice having ApoE ε3 and ε4 did not show distinguishable cognitive decline based on water maze behavior task, but when fed HFD for 6 months, mice having ApoE ε4 showed significant cognitive decline compared to mice having ApoE ε3 fed HFD, representing the importance of the brain metabolism as an environment factor to the cognition [45]. Recently, the importance of lactate to cognitive function has been reported that lactate delivered from glia via gial-neuron lactate shuttle and used as a fuel for neuronal lipid production and this lipid in a neuron are transported to glia via ApoE. Since ApoE ε4 has less efficacy to transport lipid, inability to transport lipid to glia leads to neurodegeneration [46]. Those results show the possibility that altered metabolic alteration toward glycolysis promotes lipid synthesis in neuron and induces neurodegeneration.

In the present study, we investigatedthe pyruvate metabolism of the brain in an HFD-fed mouse model using the multimodal imaging and in conjunction with the biochemical assay and the behavior test. Our results suggest that the increased hyperpolarized [1-^13^C] lactate signal in the brain of HFD-fed mice represent that altered metabolic alteration toward to glycolysis and hypoperfusion by the long-term metabolic stress by HFD further promote to glycolysis. Increased pyruvate to lactate conversion was prominent in the hippocampus and striatum which was a vulnerable area to cognitive impairment. Increased lactate signal from the brain on the hyperpolarized [1-^13^C] pyruvate MR spectroscopy could be an early sign to suggest cognitive impairment.

## Additional file

Additional file 1: **Figure S1.** Hyperpolarized [1-13C]lactate/[1-13C]pyruvate ratio in medial temporal lobe showed a negative correlation with time to spent in the target quadrant (*n* = 9, Pearson’s *r* = − 0.692, *P* < .05). (PPTX 47 kb)

## Abbreviations

Aβ: Beta amyloid
BBB: Blood brain barrier
CSI: Chemical shift image
DCE-MRI: Dynamic contrast-enhanced magnetic resonance imaging
FDG-PET: [^18^F]2-fluoro-2-deoxy-D-glucose positron emission tomography
FID: Free induction decay
GTT: Glucose tolerance test
HFD: High-fat diet
ITT: Insulin tolerance test
LOAD: Late onset Alzheimer’s disease
MCI: Mild cognitive impairment
MRS: Magnetic resonance spectroscopy
ND: Normal diet
PDH: Pyruvate dehydrogenase
PiB: Pittsburgh B
PSEN: Preseniline
