# Supplementary material for: Hyperpolarized [1-13C] pyruvate MR spectroscopy detect altered glycolysis in the brain of a cognitively impaired mouse model fed high-fat diet
Source: Mol Brain. 2018 Dec 18;11:74. doi: 10.1186/s13041-018-0415-2 (PMC6299662; doi:10.1186/s13041-018-0415-2)
Supplement: Supplementary file 1 — Figure S1. Hyperpolarized [1-13C]lactate/[1-13C]pyruvate ratio in medial temporal lobe showed a negative correlation with time to spent in the target quadrant (n = 9, Pearson’s r = − 0.692, P < .05). (PPTX 47 kb) [file 13041_2018_415_MOESM1_ESM.pptx]

## Slide 1
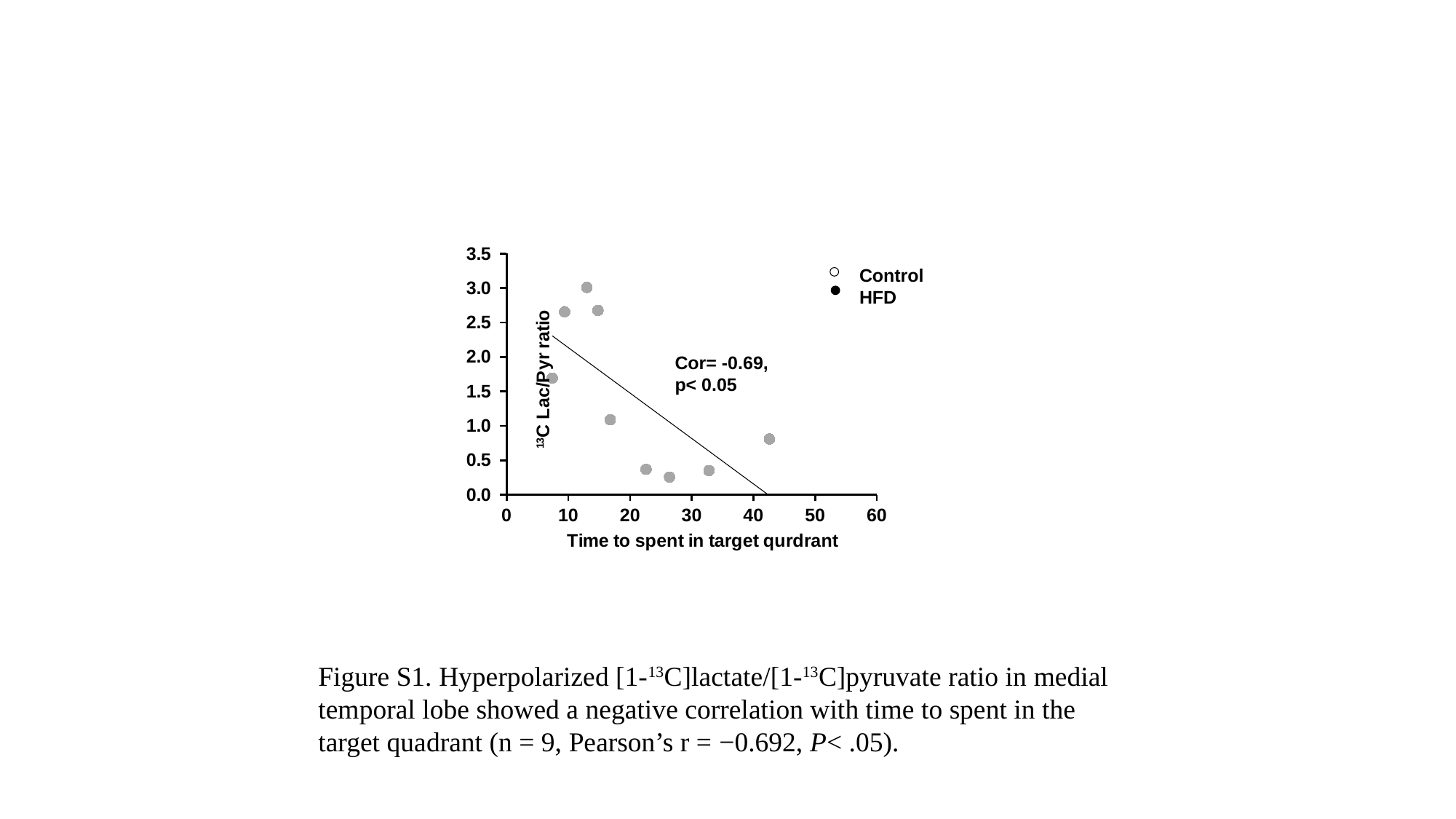

### Chart
| Category | |
|---|---|Control
HFD
Cor= -0.69,
p< 0.05
Figure S1. Hyperpolarized [1-13C]lactate/[1-13C]pyruvate ratio in medial temporal lobe showed a negative correlation with time to spent in the target quadrant (n = 9, Pearson’s r = −0.692, P< .05).
